# Acridinium 3,5-dicarboxy­benzoate monohydrate

**DOI:** 10.1107/S1600536809015529

**Published:** 2009-04-30

**Authors:** Zohreh Derikvand, Hossein Aghabozorg, Jafar Attar Gharamaleki

**Affiliations:** a Faculty of Science, Department of Chemistry, Islamic Azad University, Khorramabad Branch, Khorramabad, Iran; b Faculty of Chemistry, Islamic Azad University, North Tehran Branch, Tehran, Iran; c Faculty of Chemistry, Tarbiat Moallem University, Tehran, Iran

## Abstract

The title compound, C_13_H_10_N^+^·C_9_H_5_O_6_^−^·H_2_O, exhibits a wide range of non-covalent inter­actions, such as O—H⋯O and N—H⋯O hydrogen bonds, π–π stacking [centroid-centroid distances = 3.562 (8) and 3.872 (8) Å] and ion pairing, connecting the various components into a supra­molecular structure.

## Related literature

For background to proton transfer compounds, see: Aghabozorg *et al.* (2008 ▶); (Tabatabaee *et al.* 2009 ▶). For related structures, see: Zadykowicz, Trzybiński *et al.* (2009 ▶); Zadykowicz, Krzymiński *et al.* (2009 ▶); Trzybiński *et al.* (2009 ▶).
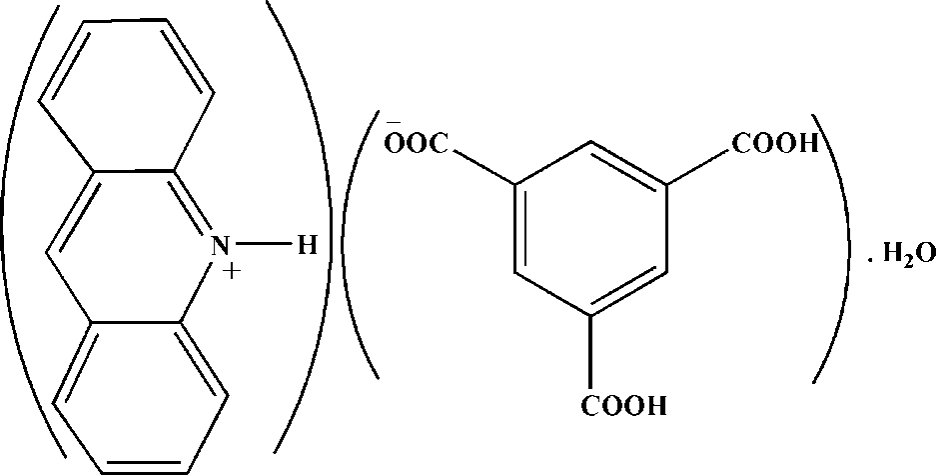


## Experimental

### 

#### Crystal data

C_13_H_10_N^+^·C_9_H_5_O_6_^−^·H_2_O
*M* *_r_* = 407.37
Triclinic, 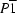

*a* = 6.8554 (4) Å
*b* = 9.6930 (6) Å
*c* = 14.8916 (10) Å
α = 103.6870 (10)°
β = 101.4240 (10)°
γ = 103.3100 (10)°
*V* = 901.62 (10) Å^3^
*Z* = 2
Mo *K*α radiation
μ = 0.11 mm^−1^
*T* = 120 K
0.25 × 0.20 × 0.15 mm

#### Data collection

Bruker SMART 1000 CCD area-detector diffractometer
Absorption correction: multi-scan (*SADABS*; Sheldrick, 1998 ▶) *T*_min_ = 0.971, *T*_max_ = 0.980
9138 measured reflections
4275 independent reflections
3659 reflections with *I* > 2σ(*I*)
*R*_int_ = 0.018

#### Refinement

*R*[*F* ^2^ > 2σ(*F* ^2^)] = 0.046
*wR*(*F* ^2^) = 0.130
*S* = 1.01
4275 reflections
271 parameters
H-atom parameters constrained
Δρ_max_ = 0.36 e Å^−3^
Δρ_min_ = −0.30 e Å^−3^

### 

Data collection: *SMART* (Bruker, 1998 ▶); cell refinement: *SAINT-Plus* (Bruker, 1998 ▶); data reduction: *SAINT-Plus*; program(s) used to solve structure: *SHELXS97* (Sheldrick, 2008 ▶); program(s) used to refine structure: *SHELXL97* (Sheldrick, 2008 ▶); molecular graphics: *SHELXTL* (Sheldrick, 2008 ▶); software used to prepare material for publication: *SHELXTL*.

## Supplementary Material

Crystal structure: contains datablocks I, global. DOI: 10.1107/S1600536809015529/pv2154sup1.cif

Structure factors: contains datablocks I. DOI: 10.1107/S1600536809015529/pv2154Isup2.hkl

Additional supplementary materials:  crystallographic information; 3D view; checkCIF report

## Figures and Tables

**Table 1:** Hydrogen-bond geometry (Å, °)

*D*—H⋯*A*	*D*—H	H⋯*A*	*D*⋯*A*	*D*—H⋯*A*
N1—H1*N*⋯O1	0.88	1.76	2.6403 (14)	174
O3—H3*O*⋯O4^i^	0.91	1.73	2.6370 (15)	174
O5—H5*O*⋯O7^ii^	0.90	1.77	2.6412 (14)	165
O7—H7*B*⋯O1	0.88	1.85	2.7197 (14)	172
O7—H7*C*⋯O2^iii^	0.88	1.94	2.7989 (15)	167

Symmetry codes: (i) 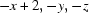; (ii) 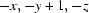; (iii) 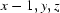.

**Fig. 1.**
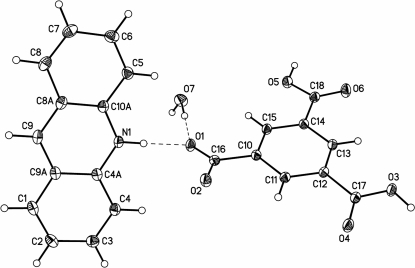
The molecular structure of the title compound, displacement ellipsoids are drawn at the 50% probability level.

**Fig. 2.**
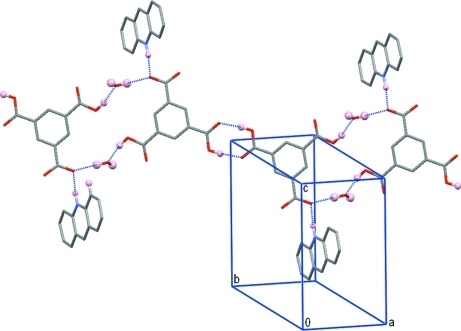
One-dimensional chain that formed by hydrogen bonds between cationic and anionic fragments and water molecules.

**Fig. 3.**
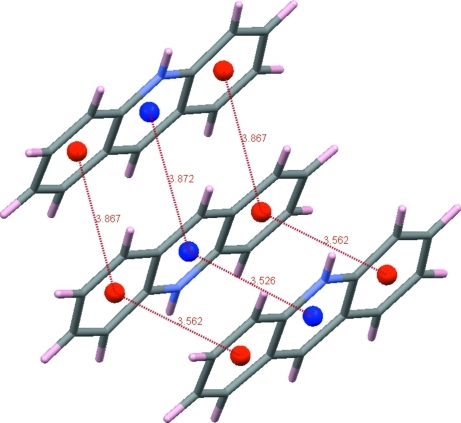
π–π Stacking interactions between cationic fragments in the title compound.

## supplementary crystallographic information

### Comment

Acridine is structurally related to anthracene wherein one of the central CH groups is replaced by nitrogen. It is a raw material used for the production of dyes and some valuable drugs. Our research group has reported the first proton transfer complex with acridine (Tabatabaee *et al.*, 2009). We have also reported many proton transfer compounds with various donor and acceptor fragments; further details and related literature has been presented in a review article (Aghabozorg *et al.*, 2008). In this article, we report the crystal structure of a proton transfer system containing acridine and benzene tricarboxylic acid.

The title structure contains a cation, an anion and a water molecule in an asymmetric unit (Fig. 1). The crystal structure shows that one of the protons of carboxylic groups has been transferred to nitrogen atom of the acridine molecule. Noncovalent interactions cause the structure to form a self-assembled system. A hydrogen bonded motif involving anion and cation fragments and water molecules linked to each other into one-dimensional chains is presented in Fig. 2; details of O–H···O and N–H···O hydrogen bonds are shown in Table 1. In addition, the interactions consisting of ion-pairing, π–π stacking [with centroid-centroid distances = 3.562 (8) and 3.872 (8) Å] between two cations are also present (Fig. 3).

The crystal structures of several acridine derivatives have been reported recently (Zadykowicz, Trzybiński *et al.*, 2009; Zadykowicz, Krzymiński *et al.*, 2009; Trzybiński *et al.*, 2009.

### Experimental

The reaction between solution benzene tricarboxylic acid (10 mg, 1 mmol) in 10 ml water and acridine (89 mg, 2 mmol) in 10 ml me thanol in 1:2 molar ratios gave brown prism crystals after slow evaporation of the solvent at room temperature.

### Refinement

All the H atoms bonded to C and N atoms were included in the refinements at idealized positions in riding motion approximation. The H atoms of the hydroxyl group and water of hydration were taken from a difference map and were not allowed to refine. The following constraints were used: C_aryl_—H = 0.95 and N—H = 0.98 Å, *U*iso(H) = 1.5*U*eq(O water) and 1.2U_eq_(the rest of the parent atoms).

### Crystal data

**Table:**

C_13_H_10_N^+^·C_9_H_5_O_6_^−^·H_2_O	*Z* = 2
*M**_r_* = 407.37	*F*(000) = 424
Triclinic, *P*1	*D*_x_ = 1.501 Mg m^−^^3^
Hall symbol: -P 1	Mo *K*α radiation, λ = 0.71073 Å
*a* = 6.8554 (4) Å	Cell parameters from 5147 reflections
*b* = 9.6930 (6) Å	θ = 2.3–30.0°
*c* = 14.8916 (10) Å	µ = 0.11 mm^−^^1^
α = 103.687 (1)°	*T* = 120 K
β = 101.424 (1)°	Prism, brown
γ = 103.310 (1)°	0.25 × 0.20 × 0.15 mm
*V* = 901.62 (10) Å^3^	

### Data collection

**Table:**

Bruker SMART 1000 CCD area-detector diffractometer	4275 independent reflections
Radiation source: fine-focus sealed tube	3659 reflections with *I* > 2σ(*I*)
graphite	*R*_int_ = 0.018
φ and ω scans	θ_max_ = 28.0°, θ_min_ = 1.5°
Absorption correction: multi-scan (*SADABS*; Sheldrick, 1998)	*h* = −9→9
*T*_min_ = 0.971, *T*_max_ = 0.980	*k* = −12→12
9138 measured reflections	*l* = −19→19

### Refinement

**Table:**

Refinement on *F*^2^	Primary atom site location: structure-invariant direct methods
Least-squares matrix: full	Secondary atom site location: difference Fourier map
*R*[*F*^2^ > 2σ(*F*^2^)] = 0.046	Hydrogen site location: mixed
*wR*(*F*^2^) = 0.130	H-atom parameters constrained
*S* = 1.01	*w* = 1/[σ^2^(*F*_o_^2^) + (0.08*P*)^2^ + 0.36*P*] where *P* = (*F*_o_^2^ + 2*F*_c_^2^)/3
4275 reflections	(Δ/σ)_max_ < 0.001
271 parameters	Δρ_max_ = 0.36 e Å^−^^3^
0 restraints	Δρ_min_ = −0.30 e Å^−^^3^

### Special details

**Table:**

Geometry. All e.s.d.'s (except the e.s.d. in the dihedral angle between two l.s. planes) are estimated using the full covariance matrix. The cell e.s.d.'s are taken into account individually in the estimation of e.s.d.'s in distances, angles and torsion angles; correlations between e.s.d.'s in cell parameters are only used when they are defined by crystal symmetry. An approximate (isotropic) treatment of cell e.s.d.'s is used for estimating e.s.d.'s involving l.s. planes.
Refinement. Refinement of *F*^2^ against ALL reflections. The weighted *R*-factor *wR* and goodness of fit *S* are based on *F*^2^, conventional *R*-factors *R* are based on *F*, with *F* set to zero for negative *F*^2^. The threshold expression of *F*^2^ > σ(*F*^2^) is used only for calculating *R*-factors(gt) *etc*. and is not relevant to the choice of reflections for refinement. *R*-factors based on *F*^2^ are statistically about twice as large as those based on *F*, and *R*- factors based on ALL data will be even larger.

### Fractional atomic coordinates and isotropic or equivalent isotropic displacement parameters (Å^2^)

**Table:**

	*x*	*y*	*z*	*U*_iso_*/*U*_eq_	
O1	0.53402 (13)	0.57945 (10)	0.27673 (6)	0.0210 (2)	
O2	0.84684 (14)	0.54218 (10)	0.30488 (6)	0.0236 (2)	
O3	0.76596 (15)	0.03275 (10)	−0.06853 (6)	0.0239 (2)	
H3O	0.8501	−0.0268	−0.0766	0.029*	
O4	0.96967 (16)	0.12545 (11)	0.08194 (7)	0.0283 (2)	
O5	0.07764 (15)	0.36662 (11)	−0.05679 (7)	0.0247 (2)	
H5O	−0.0207	0.3674	−0.1061	0.030*	
O6	0.16639 (16)	0.20983 (12)	−0.16763 (7)	0.0287 (2)	
O7	0.16191 (15)	0.62966 (12)	0.21813 (7)	0.0295 (2)	
H7B	0.2751	0.6054	0.2375	0.044*	
H7C	0.0765	0.5958	0.2499	0.044*	
N1	0.68310 (16)	0.81261 (11)	0.43083 (7)	0.0179 (2)	
H1N	0.6431	0.7352	0.3794	0.022*	
C1	0.8239 (2)	0.88639 (15)	0.69373 (9)	0.0240 (3)	
H1A	0.8669	0.9668	0.7510	0.029*	
C2	0.7957 (2)	0.74436 (16)	0.69839 (9)	0.0253 (3)	
H2A	0.8134	0.7262	0.7590	0.030*	
C3	0.7401 (2)	0.62373 (15)	0.61357 (10)	0.0241 (3)	
H3A	0.7253	0.5261	0.6184	0.029*	
C4	0.70732 (19)	0.64520 (14)	0.52498 (9)	0.0211 (3)	
H4A	0.6729	0.5637	0.4688	0.025*	
C4A	0.72531 (18)	0.79041 (13)	0.51809 (9)	0.0181 (2)	
C5	0.6491 (2)	0.96360 (15)	0.32571 (9)	0.0225 (3)	
H5A	0.6004	0.8784	0.2711	0.027*	
C6	0.6712 (2)	1.10214 (16)	0.31508 (10)	0.0264 (3)	
H6A	0.6369	1.1125	0.2525	0.032*	
C7	0.7447 (2)	1.23140 (15)	0.39604 (11)	0.0266 (3)	
H7A	0.7607	1.3267	0.3869	0.032*	
C8	0.7923 (2)	1.21908 (14)	0.48666 (10)	0.0246 (3)	
H8A	0.8408	1.3058	0.5402	0.029*	
C8A	0.76947 (18)	1.07638 (13)	0.50154 (9)	0.0197 (3)	
C9	0.81271 (19)	1.05643 (14)	0.59263 (9)	0.0214 (3)	
H9A	0.8587	1.1402	0.6481	0.026*	
C9A	0.78877 (18)	0.91388 (14)	0.60267 (9)	0.0197 (3)	
C10A	0.69968 (19)	0.94890 (14)	0.41914 (9)	0.0188 (2)	
C10	0.60515 (18)	0.40594 (13)	0.15426 (8)	0.0175 (2)	
C11	0.73036 (19)	0.31547 (13)	0.13153 (9)	0.0186 (2)	
H11A	0.8505	0.3220	0.1787	0.022*	
C12	0.68132 (19)	0.21555 (13)	0.04031 (9)	0.0180 (2)	
C13	0.50674 (19)	0.20648 (13)	−0.02982 (9)	0.0185 (2)	
H13A	0.4746	0.1400	−0.0925	0.022*	
C14	0.37953 (18)	0.29568 (13)	−0.00735 (8)	0.0179 (2)	
C15	0.42717 (18)	0.39407 (13)	0.08446 (9)	0.0180 (2)	
H15A	0.3385	0.4532	0.0996	0.022*	
C16	0.66843 (19)	0.51724 (13)	0.25273 (8)	0.0182 (2)	
C17	0.8191 (2)	0.12108 (14)	0.01994 (9)	0.0197 (2)	
C18	0.19722 (19)	0.28509 (14)	−0.08559 (9)	0.0197 (2)	

### Atomic displacement parameters (Å^2^)

**Table:**

	*U*^11^	*U*^22^	*U*^33^	*U*^12^	*U*^13^	*U*^23^
O1	0.0199 (4)	0.0232 (4)	0.0190 (4)	0.0101 (3)	0.0046 (3)	0.0011 (3)
O2	0.0210 (4)	0.0291 (5)	0.0181 (4)	0.0119 (4)	0.0008 (3)	0.0015 (4)
O3	0.0269 (5)	0.0273 (5)	0.0181 (4)	0.0167 (4)	0.0040 (4)	0.0010 (4)
O4	0.0307 (5)	0.0337 (5)	0.0201 (5)	0.0215 (4)	0.0013 (4)	0.0003 (4)
O5	0.0235 (5)	0.0319 (5)	0.0203 (4)	0.0166 (4)	0.0029 (4)	0.0047 (4)
O6	0.0282 (5)	0.0354 (5)	0.0193 (5)	0.0166 (4)	0.0011 (4)	−0.0005 (4)
O7	0.0228 (5)	0.0415 (6)	0.0279 (5)	0.0168 (4)	0.0044 (4)	0.0119 (4)
N1	0.0181 (5)	0.0184 (5)	0.0162 (5)	0.0060 (4)	0.0052 (4)	0.0019 (4)
C1	0.0213 (6)	0.0289 (6)	0.0169 (6)	0.0050 (5)	0.0031 (5)	0.0019 (5)
C2	0.0211 (6)	0.0337 (7)	0.0192 (6)	0.0067 (5)	0.0030 (5)	0.0080 (5)
C3	0.0214 (6)	0.0248 (6)	0.0252 (6)	0.0060 (5)	0.0040 (5)	0.0083 (5)
C4	0.0197 (6)	0.0205 (6)	0.0204 (6)	0.0048 (4)	0.0042 (5)	0.0029 (5)
C4A	0.0138 (5)	0.0211 (6)	0.0180 (6)	0.0046 (4)	0.0047 (4)	0.0034 (4)
C5	0.0237 (6)	0.0256 (6)	0.0202 (6)	0.0102 (5)	0.0080 (5)	0.0059 (5)
C6	0.0262 (7)	0.0314 (7)	0.0287 (7)	0.0137 (5)	0.0109 (5)	0.0142 (6)
C7	0.0233 (6)	0.0223 (6)	0.0388 (8)	0.0098 (5)	0.0121 (6)	0.0114 (6)
C8	0.0209 (6)	0.0197 (6)	0.0324 (7)	0.0076 (5)	0.0079 (5)	0.0041 (5)
C8A	0.0147 (5)	0.0193 (6)	0.0242 (6)	0.0061 (4)	0.0065 (4)	0.0028 (5)
C9	0.0187 (6)	0.0203 (6)	0.0205 (6)	0.0047 (4)	0.0047 (5)	−0.0012 (5)
C9A	0.0162 (6)	0.0217 (6)	0.0186 (6)	0.0042 (4)	0.0049 (4)	0.0022 (5)
C10A	0.0166 (5)	0.0207 (6)	0.0200 (6)	0.0070 (4)	0.0068 (4)	0.0048 (5)
C10	0.0188 (6)	0.0189 (5)	0.0160 (5)	0.0079 (4)	0.0049 (4)	0.0045 (4)
C11	0.0191 (6)	0.0195 (6)	0.0175 (5)	0.0086 (4)	0.0037 (4)	0.0042 (4)
C12	0.0197 (6)	0.0185 (5)	0.0182 (6)	0.0096 (4)	0.0065 (4)	0.0050 (4)
C13	0.0205 (6)	0.0195 (5)	0.0162 (5)	0.0085 (4)	0.0051 (4)	0.0036 (4)
C14	0.0187 (5)	0.0196 (6)	0.0165 (5)	0.0082 (4)	0.0047 (4)	0.0049 (4)
C15	0.0180 (6)	0.0189 (5)	0.0177 (5)	0.0076 (4)	0.0054 (4)	0.0040 (4)
C16	0.0201 (6)	0.0198 (5)	0.0170 (5)	0.0086 (4)	0.0055 (4)	0.0062 (4)
C17	0.0222 (6)	0.0202 (6)	0.0181 (6)	0.0103 (5)	0.0054 (5)	0.0040 (4)
C18	0.0191 (6)	0.0207 (6)	0.0199 (6)	0.0085 (4)	0.0045 (4)	0.0053 (4)

### Geometric parameters (Å, °)

**Table:**

O1—C16	1.2747 (15)	C5—C10A	1.4148 (17)
O2—C16	1.2474 (15)	C5—H5A	0.9500
O3—C17	1.3167 (15)	C6—C7	1.425 (2)
O3—H3O	0.9102	C6—H6A	0.9500
O4—C17	1.2261 (16)	C7—C8	1.364 (2)
O5—C18	1.3258 (15)	C7—H7A	0.9500
O5—H5O	0.8953	C8—C8A	1.4303 (18)
O6—C18	1.2143 (16)	C8—H8A	0.9500
O7—H7B	0.8764	C8A—C9	1.3988 (18)
O7—H7C	0.8773	C8A—C10A	1.4280 (17)
N1—C4A	1.3529 (16)	C9—C9A	1.4006 (18)
N1—C10A	1.3550 (16)	C9—H9A	0.9500
N1—H1N	0.8800	C10—C11	1.3934 (16)
C1—C2	1.366 (2)	C10—C15	1.3995 (17)
C1—C9A	1.4280 (18)	C10—C16	1.5120 (16)
C1—H1A	0.9500	C11—C12	1.3938 (16)
C2—C3	1.4188 (19)	C11—H11A	0.9500
C2—H2A	0.9500	C12—C13	1.3942 (17)
C3—C4	1.3668 (18)	C12—C17	1.4844 (16)
C3—H3A	0.9500	C13—C14	1.3950 (16)
C4—C4A	1.4144 (17)	C13—H13A	0.9500
C4—H4A	0.9500	C14—C15	1.3957 (17)
C4A—C9A	1.4275 (17)	C14—C18	1.4958 (17)
C5—C6	1.3667 (19)	C15—H15A	0.9500
			
C17—O3—H3O	111.8	C8A—C9—C9A	120.49 (11)
C18—O5—H5O	111.9	C8A—C9—H9A	119.8
H7B—O7—H7C	105.4	C9A—C9—H9A	119.8
C4A—N1—C10A	122.85 (11)	C9—C9A—C4A	118.55 (11)
C4A—N1—H1N	118.6	C9—C9A—C1	122.95 (12)
C10A—N1—H1N	118.6	C4A—C9A—C1	118.50 (12)
C2—C1—C9A	119.94 (12)	N1—C10A—C5	119.81 (11)
C2—C1—H1A	120.0	N1—C10A—C8A	119.46 (11)
C9A—C1—H1A	120.0	C5—C10A—C8A	120.73 (12)
C1—C2—C3	120.64 (12)	C11—C10—C15	119.08 (11)
C1—C2—H2A	119.7	C11—C10—C16	119.12 (10)
C3—C2—H2A	119.7	C15—C10—C16	121.77 (11)
C4—C3—C2	121.31 (12)	C10—C11—C12	120.76 (11)
C4—C3—H3A	119.3	C10—C11—H11A	119.6
C2—C3—H3A	119.3	C12—C11—H11A	119.6
C3—C4—C4A	119.08 (12)	C13—C12—C11	120.05 (11)
C3—C4—H4A	120.5	C13—C12—C17	121.31 (11)
C4A—C4—H4A	120.5	C11—C12—C17	118.63 (11)
N1—C4A—C4	119.84 (11)	C12—C13—C14	119.52 (11)
N1—C4A—C9A	119.77 (11)	C12—C13—H13A	120.2
C4—C4A—C9A	120.39 (11)	C14—C13—H13A	120.2
C6—C5—C10A	119.08 (12)	C13—C14—C15	120.32 (11)
C6—C5—H5A	120.5	C13—C14—C18	117.62 (11)
C10A—C5—H5A	120.5	C15—C14—C18	122.02 (11)
C5—C6—C7	121.30 (13)	C14—C15—C10	120.23 (11)
C5—C6—H6A	119.3	C14—C15—H15A	119.9
C7—C6—H6A	119.3	C10—C15—H15A	119.9
C8—C7—C6	120.37 (12)	O2—C16—O1	124.40 (11)
C8—C7—H7A	119.8	O2—C16—C10	118.44 (11)
C6—C7—H7A	119.8	O1—C16—C10	117.17 (10)
C7—C8—C8A	120.42 (12)	O4—C17—O3	123.27 (11)
C7—C8—H8A	119.8	O4—C17—C12	121.71 (11)
C8A—C8—H8A	119.8	O3—C17—C12	115.01 (10)
C9—C8A—C10A	118.83 (11)	O6—C18—O5	124.18 (11)
C9—C8A—C8	123.08 (12)	O6—C18—C14	122.11 (11)
C10A—C8A—C8	118.09 (12)	O5—C18—C14	113.70 (11)
			
C9A—C1—C2—C3	2.7 (2)	C8—C8A—C10A—N1	177.85 (10)
C1—C2—C3—C4	−1.9 (2)	C9—C8A—C10A—C5	178.37 (11)
C2—C3—C4—C4A	−1.30 (19)	C8—C8A—C10A—C5	−1.77 (18)
C10A—N1—C4A—C4	−179.17 (11)	C15—C10—C11—C12	0.76 (18)
C10A—N1—C4A—C9A	0.52 (18)	C16—C10—C11—C12	−177.54 (11)
C3—C4—C4A—N1	−176.65 (11)	C10—C11—C12—C13	0.80 (19)
C3—C4—C4A—C9A	3.66 (18)	C10—C11—C12—C17	−179.65 (11)
C10A—C5—C6—C7	0.2 (2)	C11—C12—C13—C14	−1.38 (18)
C5—C6—C7—C8	−0.9 (2)	C17—C12—C13—C14	179.08 (11)
C6—C7—C8—C8A	0.2 (2)	C12—C13—C14—C15	0.41 (18)
C7—C8—C8A—C9	−179.04 (12)	C12—C13—C14—C18	178.41 (11)
C7—C8—C8A—C10A	1.11 (18)	C13—C14—C15—C10	1.15 (18)
C10A—C8A—C9—C9A	0.28 (18)	C18—C14—C15—C10	−176.75 (11)
C8—C8A—C9—C9A	−179.57 (11)	C11—C10—C15—C14	−1.73 (18)
C8A—C9—C9A—C4A	1.79 (18)	C16—C10—C15—C14	176.53 (11)
C8A—C9—C9A—C1	−177.87 (11)	C11—C10—C16—O2	11.75 (17)
N1—C4A—C9A—C9	−2.23 (17)	C15—C10—C16—O2	−166.50 (11)
C4—C4A—C9A—C9	177.46 (11)	C11—C10—C16—O1	−168.22 (11)
N1—C4A—C9A—C1	177.44 (11)	C15—C10—C16—O1	13.52 (17)
C4—C4A—C9A—C1	−2.87 (18)	C13—C12—C17—O4	−177.96 (12)
C2—C1—C9A—C9	179.34 (12)	C11—C12—C17—O4	2.49 (19)
C2—C1—C9A—C4A	−0.32 (19)	C13—C12—C17—O3	1.45 (17)
C4A—N1—C10A—C5	−178.76 (11)	C11—C12—C17—O3	−178.10 (11)
C4A—N1—C10A—C8A	1.62 (18)	C13—C14—C18—O6	−4.89 (18)
C6—C5—C10A—N1	−178.51 (11)	C15—C14—C18—O6	173.07 (12)
C6—C5—C10A—C8A	1.10 (19)	C13—C14—C18—O5	176.13 (11)
C9—C8A—C10A—N1	−2.01 (17)	C15—C14—C18—O5	−5.91 (17)

### Hydrogen-bond geometry (Å, °)

**Table:**

*D*—H···*A*	*D*—H	H···*A*	*D*···*A*	*D*—H···*A*
N1—H1N···O1	0.88	1.76	2.6403 (14)	174
O3—H3O···O4^i^	0.91	1.73	2.6370 (15)	174
O5—H5O···O7^ii^	0.90	1.77	2.6412 (14)	165
O7—H7B···O1	0.88	1.85	2.7197 (14)	172
O7—H7C···O2^iii^	0.88	1.94	2.7989 (15)	167

Symmetry codes: (i) −*x*+2, −*y*, −*z*; (ii) −*x*, −*y*+1, −*z*; (iii) *x*−1, *y*, *z*.
